# The practice of breast self-examination and associated factors among female healthcare professionals working in selected hospitals in Kigali, Rwanda: a cross sectional study

**DOI:** 10.1186/s12905-023-02776-4

**Published:** 2023-11-23

**Authors:** Mulugeta Tenna Wolde, Rosemary Okova, Michael Habtu, Mekitie Wondafrash, Abebe Bekele

**Affiliations:** 1 Department of Epidemiology and Biostatistics, School of Public Health, Mount Kenya University, Kigali Campus, Kigali, Rwanda; 2 School of Nursing, Mount Kenya University, Kigali Campus, Kigali, Rwanda; 3 College of Medicine and Health Sciences, School of Public Health, University of Rwanda, Kigali, Rwanda; 4 St. Paul Institute for Reproductive Health and Rights, Addis Ababa, Ethiopia; 5 University of Global Health Equity, Kigali, Rwanda

**Keywords:** Breast self-examination, Breast cancer, Female healthcare professionals

## Abstract

**Background:**

Breast self-examination (BSE) is considered one of the main screening methods in detecting earlier stages of breast cancer. It is a useful technique if practiced every month by women above 20 years considering that breast cancer among women globally contributed to 685,000 deaths in 2020. However, the practice of breast self-examination among healthcare professionals is low in many developing countries and it is not well known in Rwanda. Therefore, this research was intended to measure the level of breast self-examination practice and its associated factors among female healthcare professionals working in selected hospitals in Kigali, Rwanda.

**Methods:**

A cross-sectional study was conducted among 221 randomly selected female healthcare professionals in four district hospitals in Kigali, Rwanda. A self-administered structured questionnaire was used as data collection instrument. The predictor variables were socio-demographic and obstetrics variables, knowledge on breast cancer and breast self-examination as well as attitude towards breast cancer and breast self-examination. Sample statistics such as frequencies, proportions and mean were used to recapitulate the findings in univariate analysis. Multiple logistic regression analysis was employed to identify statistically significant variables that predict breast self-examination practice. Adjusted odds ratio with 95% confidence level were reported. *P*-value < 0.05 was used to declare statistical significance.

**Results:**

Breast self-examination was practiced by 43.5% of female healthcare professionals. This prevalence is low compared to other studies. Attitude towards breast self-examination and breast cancer was the only predictor variable that was significantly associated with breast self-examination practice [AOR = 1.032; 95% CI (1.001, 1.065), *p*-value = 0.042]. However, number of pregnancy and number of children were not significantly associated with BSE practice in the multi-variate analysis. In addition, there was a positive linear link between knowledge and attitude, with a correlation coefficient (r) of 0.186 (*p* = 0.005).

**Conclusions:**

The breast self-examination practice among healthcare professionals was found to be low. Attitude towards breast cancer and breast self-examination was positively associated with BSE practice. Moreover, attitude and knowledge were positively correlated. This suggests the need for continuous medical education on breast self-examination and breast cancer to increase the knowledge & BSE practice level of female healthcare professionals.

## Background

Globally, an estimated 19.3 million new cancer cases and around 10.0 million cancer related deaths were reported in 2020 [1]. According to the International Agency for Research on Cancer (IARC), in 2020, female breast cancer (BC) superseded lung cancer as the most commonly diagnosed cancer, with an estimated incidence of 2.3 million new cases (11.7%), followed by lung (11.4%), colorectal (10.0%), prostate (7.3%), and stomach (5.6%) cancers and breast cancer is the fifth leading cause of cancer mortality worldwide, with 685,000 deaths in 2020 [1]. Almost 99% of breast cancer affects females, and only 1% of men are affected by breast cancer.

Among women globally, breast cancer accounts for 1 in 4 cancer cases and 1 in 6 cancer deaths, ranking first for cancer incidences in 159 among 185 countries and for mortality in 110 countries [1]. Though the incidence of female breast cancer is low in Africa, its incidence has been on the rise in the last decades. Moreover, the mortality rate due to breast cancer is higher in developing counties than developed countries [2].

The Globocan 2020 data revealed that breast cancer and cervix uteri cancers are the two most commonly diagnosed and leading causes of cancer related mortality in Rwanda. It contributed to 24% of all new cancer cases diagnosed in Rwanda [3].

A retrospective study done in Rwanda has shown that there is a delay in diagnosing breast cancer patients and most cases present in advanced stages at the start of treatment [4]. Moreover, there is only one center of excellence of Butaro Hospital located in Burera District in Northern Province for treatment of all cancer patients. It is only recently that radiotherapy machines are installed in Rwanda Military Hospital (RMH) [5].

One of the ways of improving survival rates of women with BC is early detection through screening programs [6]. The three commonly practiced approaches of BC screening are clinical breast examination (CBE), mammography and breast self-examination (BSE). BSE is the most practical and useful screening method in resource scarce Sub-Saharan Africa (SSA) countries [7] where other screening methods are not widely available and practiced [8–10].

Breast self-examination is a simple, less costly physical examination done by a woman on a monthly basis privately to detect changes in texture, color, and size of her breast and also identify swelling and lumps so that earlier stages of the disease are referred to the hospital for treatment [11]. The American Cancer Society (ACS) recommends that women to be cognizant of their breast look and feel and should report to a healthcare provider right away whenever they detect any abnormality [12].

According to the study carried out by Johns Hopkins Medical center, 40 % (40%) of breast cancers are diagnosed after females detecting a lump during routine breast self examination [13]. Although there is still a controversy about the effectiveness of BSE in reducing cancer related mortality, WHO recommends to combine BSE with other screening methods in detecting BC at earlier stage [6].

It is reported that women in low and middle income countries don’t practice BSE widely [14]. Moreover, it is negligible percentage of women who have practiced BSE with the correct procedure and timing in most African countries [15].

One scoping review has revealed that BSE practice was still low in SSA countries [16]. Another similar study has also shown the low prevalence of BSE practice in most African countries despite a relatively high awareness level [11].

Female healthcare professionals are key influential figures to change the attitude, beliefs and behaviors of the general public in the community to practice BSE. It is also expected that healthcare professionals widely practice BSE. However, many studies have shown that the level of BSE practice was found to be low in many developing countries. A study done in Saudi Arabia has shown that 74.7% of healthcare professionals practicing BSE [17]. Similar prevalence was also observed in a study carried out among nurses in Eritrea, 75.5% [18]. A meta-analysis study has revealed a lower proportion of women practicing BSE among healthcare professionals in Ethiopia, 56.3% [19]. This is in conformity with the ever prevalence of BSE practice among healthcare professionals in another study done in Ethiopia, 53% [20]. A low level of BSE practice was also reported among Iranian healthcare professionals that showed only 9% of them practiced on monthly basis [21]. This shows that there is no uniformity in the BSE practice among healthcare professionals.

From the literatures reviewed, it can be revealed that knowledge, attitude, family history of BC and some socio-demographic characteristics (level of education, socio-economic status, and age) were regarded as good predicators for BSE practice. However, the association is not consistent as shown in the different studies. For example, the predicted variables for practicing BSE were having high level of knowledge, good attitude and having breast cancer in the family as evidenced in a study done in Africa, [9]. To the contrary, there was no statistically significant association between attitude and BSE practice as shown in the study done among female healthcare workers in Debre Tabor Town, Northern part of Ethiopia [22]. Another predicators were personal history of BC and getting to know someone with BC [20].

There is a paucity of literature on BSE practice in Rwanda especially among healthcare professionals. Thus, the purpose of this study was to assess the practice of BSE and factors associated with BSE practice among female healthcare professionals working in selected hospitals located in Kigali, Rwanda.

## Materials and methods

### Study design

An institution based cross-sectional study design was employed to collect data from four district hospitals in Kigali, the capital city of Rwanda from October to December 2022. The hospitals are located in the three administrative districts of Kigali namely Gasabo (Kacyiru and Kibagabaga hospitals), Nyarugenge (Muhima hospital) and Kicukiro (Masaka hospital). All the four hospitals are district level hospitals as per the Ministry of Health (MoH) system and they provide curative and rehabilitative services for patients referred from primary level health facilities. In addition, the hospitals are responsible to monitor the promotional and preventative services provided at the primary health level facilities. The number of beds in the hospitals varies from 208 to 250 with bed occupancy rate of 90–95%.

### Study population

All female healthcare professionals aged above 20 years were eligible to be enrolled in this study. However*,* female healthcare professionals who had undergone a bilateral mastectomy procedure and those who declined to consent were excluded from this study.

### Sample size & sampling techniques

There were a total of 577 female healthcare workers in the four district hospitals involved in the study. The list of female healthcare professionals was received from the Human Resource Departments of the four district hospitals to create the sampling frame for each hospital. The number of female healthcare professionals in Kacyiru, Kibagabaga, Masaka and Muhima were 110, 149, 158, and 160 respectively. A simple random probability sampling technique using randomly generated numbers in excel was utilized to select 242 samples proportinalte to the size of the study population in each hospital (46 samples from Kacyiru, 63 from Kibagabaga, 66 from Masaka, and 67 from Muhima hospitals), of which 221 female healthcare workers have returned the questionnaires. A single population proportion formula was used with assumption of a 5% significance level, a 5% precision and prevalence of 56.31% taken from a study conducted in Ethiopia [19]. The 10% non-response rate and a finite population correction factor were considered to reach the final sample size of 242.

### Data quality & collection procedure

Reliability of the data collection instrument was ensured as the Cronbach’s alpha coefficient for the constrct variables knowledge, attitude and practice were 0.84, 0.79 and 0.95 respectively [23].

The data collection instrument was a pre-tested self-administered structured questionnaire. It was adapted following a detailed review of the literatures and modified to fit to this study [17]. It was administered in English as most healthcare professionals in Rwanda were able to understand the language. Data collected included socio-demographic features, gynecological/obstetrics history, knowledge on BC and BSE, attitudes towards BSE and BC, practice of BSE. The only outcome variable was BSE practice while all the other variables were considered as predictor variables.

This study was approved by Ethical Review Board and Institue of Post-gradaute studies and Research of Mount Kenya University before contacting the hospitals for data collection.

The informaitona and data have been kept confidential. Participation in this study was voluntary, and the participants could discontinue participation at any time and for any reason.

### Operational definitions

*Attitudes*: perception of the female healthcare professionals towards BSE & BC as measured by 19 elements of attitude with five points Likert scale. High score indicates a favorable attitude.

*Good BSE Practice*: a score equal to or above 7 out of 13 “Yes” or “No” questions.

*Knowledge on BC & BSE*: was defined as knowledge of female healthcare professionals on BSE & BC as measured by structured knowledge questions. A high score indicates a higher knowledge.

*Female healthcare professionals*: include health professionals working in the different departments of the hospital who are holders of high school and advanced diplomas, bachelor, masters or PhD. It doesn’t include helpers and those in charge of medical waste handling. High school diploma refers to A2 level who are registered nurses with general nursing training during a secondary education while advanced diploma refers to A1 level who are registered nurses with completed 3 years of general nursing training in a post-secondary education setting.

### Data management & analysis technique

The collected data was checked for completeness and each questionnaire was coded. The paper based data was entered into Epidata v4.6 and then exported into IBM SPSS v.26 for data management, cleaning, and analysis. The total score for knowledge, attitudes and practice was calculated for each respondent where by the variables knowledge and attitude were considered as continuous variables while the construct BSE practice was considered as a categorical variable after dichotomization into Good practice and Poor practice.

Knowledge was assessed in four dimensions of knowledge on BSE, BC risk factors, signs and symptoms of BC and methods of diagnosis. Each item was evaluated as ‘Yes’, ‘No’ and ‘I don’t know’. Score one [1] was given to the correct answers and zero (0) for incorrect and “I don’t know” answers [17, 18]. The dimension of knowledge on BSE had 7 items with total score ranging from 0 to 7, knowledge on BC risk factors had 13 elements with total score of 0–13, BC signs and symptoms had 10 elements with a total score of 0–10 and methods of diagnosis of BC has 5 elements with a total score of 0–5. Therefore, the overall score for knowledge ranged from 0 to 35. The percent score for the total knowledge was calculated for each study participant.

There were 19 elements for the construct attitude measured on 5-points Likert scale with a total score ranging from 19 to 95. The percent score for attitude was calculated for each study participant. Ten negatively worded items/questions in the construct attitude were reversely coded before adding the total score.

The section on BSE practice included whether the participant ever practiced BSE or not. It also included if the BSE practice was done on regular basis and as per the recommended timing and technique. The total score for BSE practice was out of 13. This variable was dichotomized as good/regular practice if the total score for BSE practice of the respondent was equal and above 7 and poor/irregular practice if the total score was below 7 [24].

Descriptive statistics such as frequencies and percentages were used to summarize the results of categorical variables while mean/standard deviation (SD) and medians were used to summarize the findings of continuous variables in univariate analysis. Normality test was done using Shapiro-Wilk test for knowledge and attitude scores. Mann Whitney U and Kruskal Wallis tests were used to compare knowledge score as the data was not normality distributed. However, comparison for attitude was done using independent t-test and ANOVA test as the data was normally distributed.

Bivariate analysis between each independent and the BSE practice was done in binary logistic regression model and those variables that showed significant association with BSE practice with *p*-value of < 0.25 were entered into the multivariate analysis to control for possible confounding variables and create the best fit model for prediction of the dependent variable which is practice of BSE. In multivariable binary logistic regression, adjusted odds ratio (AOR) with a 95% CI and *p*-values < 0.05 were considered to identify statistically significant predicators in the final model [25].

Hosmer and Lemeshow test was used to assess the goodness of fit of the data and the *p*-value was found to be 0.73 which indicates the model adequately fitted the data. The assumption of no multi-collinearity among the continuous predictor variables was checked and the Variance Inflation Factor (VIF) was less than 5 for most of the variables that showed there was no multi-collinearity.

## Results

### Socio-demographic characteristics

A total of 242 female healthcare professionals were randomly selected and 221 returned the questionnaires with a response rate of 91.3%. The age of the respondents ranged from 22 to 58 years with mean age of 35.3 ± (SD 7.1). Close to 45 % of respondents 99 (44.8%) were in the age group 31–40 years of age. Of the 221 respondents, 177 (80.1%) were nurses, 10 (4.5%) were doctors and 34 (15.4%) were other medical professionals. More than half of them 127 (57.5%) were holders of advanced A1 diplomas. About 85 (38.5%) were followers of Catholic religion followed by Protestant 79 (35.7%) and Adventist 30 (13.6%). Almost three-fourth of the study population 165 (75.1%) were married. Nearly one third of the participants 77 (34.8%) had more than 10 years of work experience. Almost all of the participants 214 (98.6%) are in the Rwandan socio-economic class (Ubudehe) category III (Table 1).

**Table 1:** Socio-demographic characteristics of female healthcare professionals in four district hospitals in Kigali Rwanda, 2022 (*n* = 221)

Variables	Frequency	Percent
**Age (years)**		
21–30	70	31.7
31–40	99	44.8
Above 41	52	23.5
**Profession**		
Nurse	177	80.1
Doctor	10	4.5
Other	34	15.4
**Level of education**		
A2 (High-school diploma)^a^	11	5.0
A1 (2–3 years college diploma)^b^	127	57.5
Bachelor degree & above	83	37.6
**Socio-economic category**^**c**^		
Category III	218	98.6
Category IV	3	1.4
**Religion**		
Adventist	30	13.6
Catholic	85	38.5
Muslim	6	2.7
Protestant	79	35.7
Other	21	9.5
**Marital status**		
Single	48	21.7
Married	166	75.1
Divorced & Widowed	7	3.2
**Work Experience**		
<10 years	144	65.2
≥10 years	77	34.8

^a^A2 refers to high school nursing diploma who are registered and trained with general nursing during a secondary education

^b^A1 refers to advanced diploma nursing who are registered and trained with completed 3 years of general nursing in a pot-secondary education setting

^c^Category III means self-sustaining while category IV is for those who rich [26]

### Obstetrics and breast cancer history of female healthcare professionals

Most of the respondents 162 (73.3%) had history of pregnancy with mean age at first pregnancy being 25.9 years ± (SD 3.5) and the average number of pregnancy and children were 2.8 ± (SD 1.5) and 2.6 ± (SD 1.2) respectively. The average duration of breastfeeding of the last child was 1.9 ± (SD 0.89) years. Minority of the participants had breast cancer history at personal level 17 (7.7%), at the levels of first degree relatives 10 (4.5%) and second degree relatives 8 (3.6%). Out of a total 54 (24.4%) participants who received training on BSE, 41.5% received on-the-job training while 35.9% at school and 22.6% in both school and on-the-job (Table 2).

**Table 2:** Obstetrics and breast cancer history of female healthcare professionals, Kigali, Rwanda, 2022, (*n* = 221)

Variables	Mean	SD	Frequency	Percent
**Age at first pregnancy** ^a^ **(*n*** **= 162)**^b^	25.88	3.50		
**Number of children** ^a^	2.60	1.24		
**Number of pregnancy** ^a^	2.83	1.47		
**Duration of breastfeeding** ^a^	1.88	.89		
**History of self-breast cancer**				
No			204	92.3
Yes			17	7.7
**History of breast cancer in 1st relative**				
No			211	95.5
Yes			10	4.5
**History of breast cancer in 2nd relative**				
No			213	96.4
Yes			8	3.6
**Training on BSE**				
No			167	75.6
Yes			54	24.4
**Time of BSE training received**				
In the higher institution			19	35.8
On-the-job			22	41.5
In the higher institution and on-the-job training			12	22.6
**Name of the hospital**				
Kacyiru hospital			36	16.3
Kibagabaga hospital			63	28.5
Masaka hospital			56	25.3
Muhima hospital			66	29.9

^a^ Mean and standard deviation was calculated for continuous variables. *SD* Standard Deviation

^b^ The total number of study participatns who have ever been pregnant (*n* = 162)

### Knowledge about breast cancer and breast self-examination

The knowledge level of participants on the correct age of commencement of BSE practice was 56.6% and slightly over two-thirds of the participants 150 (67.9%) knew that BSE has to be done on monthly basis. However, only less than half of the participants 96 (43.4%) knew the recommended timing to do BSE which is 3–5 days after the cession of the monthly menses. The majority of respondents 190 (86.0%) acknowledged that BSE is used for early detection of BC and 195 (88.2%) have affirmed that it is done by inspection and palpation and 88.2% replied that a woman had to consult a doctor within few days after detecting the mass. However, the majority 177 (80.1%) wrongly reported that a healthcare professional had to wait for the progress of the mass for few months before consulting a doctor (Table 3).

**Table 3:** Participants correct responses on knowledge of BSE & BC questions, *n* = 221

Variables	Frequency	Percent
**Knowledge BSE**		
Age to start BSE is at 20 years	125	56.6
A woman ought to examine her breast on monthly basis	150	67.9
The appropriate time for BSE is during the monthly menses	96	43.4
BSE helps in detecting early stage of breast cancer	190	86.0
BSE is performed by inspection and palpation	195	88.2
Consult a doctor within few days of finding a mass	195	88.2
A health professional has to follow the growth of the mass	44	19.9
**Knowledge on risk factors**		
Family history with breast cancer	187	84.6
High fat diet	128	57.9
Smoking	178	80.5
Prolonged breastfeeding	122	55.2
Having the first child after 35 years of age	95	42.9
Early menarche	68	30.8
Having late menopause	78	35.3
Being obese	111	50.2
Nulliparity	86	38.9
Marital status	41	18.6
No breast feeding	86	38.9
Contagious from other people	159	71.9
Breast surgery for reasons other than BC	90	40.7
**BC symptoms**		
Breast lump	157	71.0
Breast pain	186	84.2
Breast swelling	187	84.6
Nipple discharge	176	79.6
Change in the color of the breast	179	81.0
Dimpling of the breast	152	68.8
Ulcer on the breast	162	73.3
Weight loss	133	60.2
A lump under the armpit	145	65.6
Scaling/dry skin in nipple region	143	64.7
**Methods of diagnosis**		
Mammography	196	88.7
Breast ultrasound	162	73.3
Breast self-examination	26	11.8
FNA/Biopsy	194	87.8
CBE	27	12.2

The commonly recognized risk factors by female healthcare professionals were family history of breast cancer (84.6%) and smoking (80.5%). Nulliparity, having first child at older age, early menarche and late menopause were known by less than half of the participants. About 55% stated that prolonged breastfeeding is not a risk factor for BC (Table 3).

Most of the symptoms of BC were known by female healthcare professionals except weight loss that was mentioned by only 60.2% of participants. Breast pain (84.2%) and swelling (84.46%) were the most commonly recognized symptoms of breast cancer (Table 3).

Most respondents knew diagnostic mammography (88.7%), breast ultrasound (73.3%) and biopsy (87.8%) as methods of diagnosis of breast cancer. However, only 12.2 and 11.8% of the respondents had knowledge on CBE and BSE as screening methods rather than diagnostic tools respectively (Table 3).

The overall median knowledge score of respondents was 62.9%, and only 44.8% of the study participants scored above the median. The median score of the participants on BSE knowledge was 71.4% while the median score of respondents on BC risk factors, symptoms and methods of diagnosis were 46.2, 80.0 and 60.0% respectively.

**Fig. 1:**
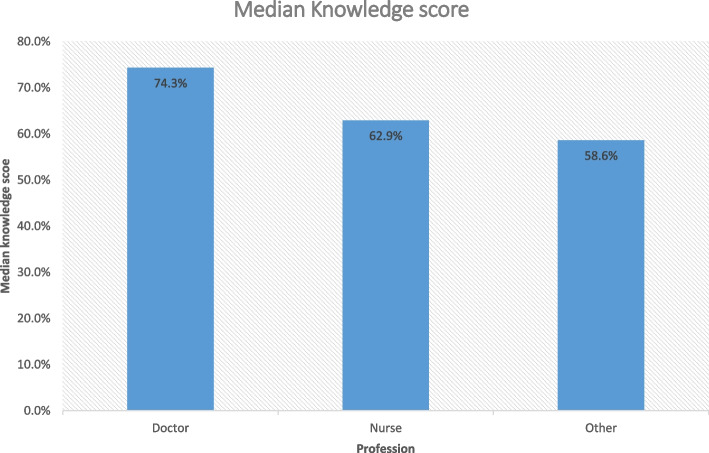
Median knowledge score among different professions, 2022

Female doctors had statistically significant higher knowledge level than nurses and other health professionals with median score of 74.3% compared to 62.9% for nurses and 58.6% for other professions, *p*-value = 0.011 (Fig. 1).

Holders of Bachelor degree (A0) had statistically significant higher total knowledge score than those with advanced diploma (A1), *p* = 0.034.

Female healthcare professionals who received training on BSE either on the job or school had statistically significant higher total knowledge score than those without training, *p*-value = 0.007.

There was no statistically significant knowledge difference between the groups with good BSE practice and poor BSE practice with *p*-value = 0.064.

### Attitude of respondents to breast self-examination

The mean for the total attitude score toward BSE and BC was 72.8 ± (SD 11.1). Table 4 shows the frequency distribution of the 19 items used to measure attitude in 5-points Likert’s scale.

**Table 4:** Participants’ response to attitude towards BC and BSE, *n* = 221

Attitude Questions	Likert’s scale				
	Strongly disagree	Disagree	Neutral	Agree	Strongly agree
BC does not occur in the Old	60 (27.1)	70 (31.7)	36 (16.3)	37 (16.7)	18 (8.1)
Family history is a risk for BC	25 (11.3)	22 (10.0)	22 (10.0)	91 (41.2)	61 (27.6)
Advanced BC is not curable	29 (13.1)	38 (17.2)	40 (18.1)	86 (38.9)	28 (12.7)
BSE is a very useful method in detecting breast cancer	21 (9.5)	13 (5.9)	21 (9.5)	101 (45.7)	65 (29.4)
BSE should be promoted nationwide	20 (9.0)	13 (5.9)	28 (12.7)	95 (43.0)	65 (29.4)
I will teach women on BSE	16 (7.2)	23 (10.4)	29 (13.1)	98 (44.3)	55 (24.9)
Any woman can do BSE	21 (9.5)	25 (11.3)	21 (9.5)	94 (42.5)	60 (27.1)
There is a huge benefit from doing regular BSE	20 (9.0)	19 (8.6)	26 (11.8)	96 (43.4)	60 (27.1)
There are limited barriers to practice BSE	32 (14.5)	52 (23.5)	28 (12.7)	85 (38.5)	24 (10.9)
BSE isn’t pleasant practice	42 (19.0)	77 (34.8)	40 (18.1)	42 (19.0)	20 (9.0)
Early diagnosis prolongs a woman’s life	16 (7.2)	24 (10.9)	25 (11.3)	97 (43.9)	59 (26.7)
I believe that I can’t identify any abnormality	42 (19.0)	81 (36.7)	37 (16.7)	43 (19.5)	18 (8.1)
There is no reason to do breast self-examination	104 (47.1)	72 (32.6)	19 (8.6)	17 (7.7)	9 (4.1)
If there is no abnormality detected during BSE, there is no need to do mammography	50 (22.6)	100 (45.2)	21 (9.5)	35 (15.8)	15 (6.8)
Early detection methods for breast cancer have no effect on treatment	63 (28.5)	83 (37.6)	24 (10.9)	38 (17.2)	13 (5.9)
BSE is very difficult for illiterate women,	59 (26.7)	87 (39.4)	43 (19.5)	22 (10.0)	10 (4.5)
BC is non curable	52 (23.5)	104 (47.1)	34 (15.4)	18 (8.1)	13 (5.9)
Only educated women can do BSE	78 (35.3)	80 (36.2)	22 (10.0)	27 (12.2)	14 (6.3)
BC does not occur in young	72 (32.6)	98 (44.3)	27 (12.2)	14 (6.3)	10 (4.5)

Three quarter of the respondents 166 (74.1%) have agreed BSE as a useful method to detect breast cancer. The majority of the participants 160 (72.4%) agreed that BSE has to be promoted at the national level. Nearly two third of the respondents 154 (64.9%) believed that BSE could be done by any woman. However, only 123 (55.7%) were confident that they could identify abnormality during BSE.

Knowledge on BC and BSE had a positive linear relationship with attitude toward BSE with r = 0.186, *p* = 0.005.

### Breast self-examination practice of respondents

Less than half of the female health care professionals 94 (42.5%) have ever practiced BSE. Moreover, only 73 (33.0%) respondents have shown to have a Good/Regular practice. Only 56.4% of female healthcare professionals conducted BSE on monthly basis, and less than half of them (47.9%) practiced as per the recommended timing. Less than half of the participants (48.9%) reported to practice BSE in lying down position. However, more than 70% of participants reported to examine the arm pit, apply different pressures and follow the recommended pattern during BSE practice (Table 5).

**Table 5:** BSE Practice among healthcare professionals, Kigali, Rwanda, 2022

**Variable**	**Ever practiced (*n*** **= 221)**	
	**No (frequency/%)**	**Yes (frequency/%)**
Ever practiced BSE	127 (57.7)	94 (42.5)
**Variables**	**Correct Responses (*n*** **= 94)**	
	**No (frequency/%)**	**Yes (frequency/%)**
Practiced BSE on monthly basis	41 (43.6)	53 (56.4)
Practiced BSE few days after menses ends	49 (52.1)	45 (47.9)
Practiced BSE by standing in front of a mirror	33 (35.1)	61 (64.9)
Practiced BSE during taking shower	47 (50.0)	47 (50.0)
Practiced BSE in lying down position	48 (51.1)	46 (48.9)
Practiced BSE using the pads of three middle fingers	31 (33.0)	63 (67.0)
Examined armpit during BSE	20 (21.3)	74 (78.7)
Followed a circular or up and down pattern during BSE	14 (14.9)	80 (85.1)
Palpated the neck region during BSE	36 (38.3)	58 (61.7)
Applied different level of pressure during BSE	26 (27.7)	68 (72.3)
Squeezed the nipple during BSE	28 (29.8)	66 (70.2)
Recorded the findings of BSE on a notebook	45 (47.9)	49 (52.1)

**Fig. 2:**
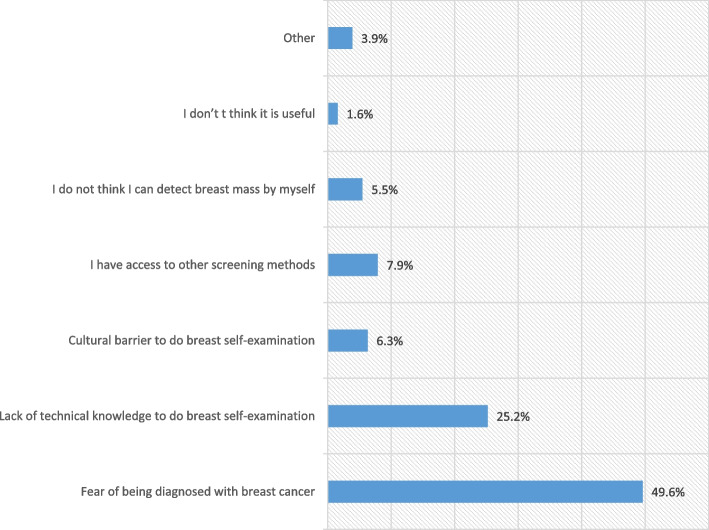
Reasons for not practicing BSE among study participants, Kigali hospital, Rwanda, 2022

Fear of being diagnosed with BC was the major reason for not practicing BSE in almost half of the participants (49.6%). A quarter of the participants (25.2%) have mentioned lack of technical knowledge as a second reason hindering BSE Practice (Fig. 2).

### Predictors of breast self-examination practice

The bivariate logistic analysis has showed that age, religion, number of pregnancy, number of children, duration of breastfeeding, training on BSE, knowledge, attitude and work experience were the candidate variables for multivariate logistic regression analysis at *p*-value of < 0.25.

The multivariate analysis result revealed that attitude had a significant positive impact on BSE Practice after adjusting for confounding, [AOR = 1.032; 95% CI (1.001, 1.065)]. However, other candidate variables were not found to be good predictors of BSE practice (Table 6).

**Table 6:** Bivariate and Multivariate Logistic regression for association between BSE and Predictors of BSE among healthcare professionals in Kigali, Rwanda, 2022, (*n* = 221)

Variables	BSE Practice		COR(95%CI)	AOR(95%CI)	*P*-value
	No	Yes			
**Age (years)** ^**a**^			1.023 (0.99–1.07)	0.98 (0.91–1.06)	0.585
**Religion**					
Adventist	18 (14.2%)	12 (12.8%)	1	1	
Catholic	39 (17.6%)	46 (20.8%)	1.09 (0.46–2.59)	1.08 (0.43–2.67)	0.287
Muslim	5 (2.3%)	1 (0.5%)	0.34 (0.04–3.35)	0.33 (0.32–3.44)	0.875
Protestant	50 (22.6%)	29 (13.1%)	0.80 (0.33–1.93)	0.76 (0.30–1.94)	0.354
Other	15 (6.8%)	6 (2.7%)	0.29 (0.07–1.20)	0.29 (0.06–1.273)	0.100
**Number of pregnancy** ^**a**^			1.22 (1.04–1.43)	1.23 (0.72–2.12)	0.447
**Number of children** ^**a**^			1.23 (1.03–1.47)	0.91 (0.47–1.76)	0.788
**Duration of breastfeeding** ^**a**^			1.22 (0.95–1.56)	1.03 (0.73–1.45)	0.868
**Training on BSE**					
No	100 (45.2%)	67 (30.3%)	1	1	
Yes	27 (12.2%)	27 (12.2%)	1.74 (0.92–3.27)	1.47 (0.74–2.91)	0.276
**Knowledge Total** ^**a**^			1.04 (0.99–1.09)	1.02 (0.97–1.08)	0.442
**Attitude Total** ^**a**^			1.03 (1.001–1.06)	1.03 (1.00–1.07)	0.042*
**Work experience**					
<10 years	88 (39.8%)	56 (25.3%)	1	1	
≥10 years	39 (30.7%)	38 (40.4%)	1.53 (0.88–2.68)	1.24 (0.46–3.32)	0.668

*COR* Crude Odds ratio, *AOR* Adjusted Odds Ration, 1 = Reference category

* indicates statistical significant at *p* < 0.05

^a^ continuous variable

## Discussion

The present study was conducted to assess the prevalence of BSE and its associated factors among female healthcare professionals working in selected district hospitals in Kigali, Rwanda.

There are four important findings in this study. The first finding was the low level of BSE practice. The second findingwas more than 50% of female healthcare professionals didn’t mention the commonly known risk factors of breast cancer. The third finding revealed that almost half of the participants felt that they are unable to detect breast cancer by themselves. The last finding showed only attitude as a predicator to practice BSE.. Attitude was the only statistically significant predictor for BSE practice.

In this study, less than half of female healthcare professionals, 94 (42.5%) reported ever performing BSE, yet regular performers were only 33.0%. The main reasons for not practicing BSE was fear of being diagnosed with breast cancer followed by lack of technical knowledge to perform BSE. Similar prevalence of regular BSE practice was observed in prior study conducted among female healthcare professionals in Oromia region of Ethiopia, 32.6% [27]. However, the prevalence of BSE practice in this study was lower than previous studies done in Saudi Arabia, Ethiopia, Eritrea, Turkey, Nigeria and Morocco [17, 18, 20, 28–32]. The possible explanations for this difference could be differences in educational level of participants, size and composition of the samples, access to information and possible increased breast cancer awareness campaigns. For example, most of the respondents were holders of bachelor degree and the proportion of doctors was higher in the study done by Heena in Saudi Arabia [17].

The magnitude of breast self-examination practice in this study was higher compared to a study done in North West Ethiopia [22]. The study participants being young and living in relatively rural area might have an impact in the magnitude of BSE practice in Ethiopia.

Moreover, the prevalence of BSE practice in this study was higher compared to the study done among secondary students in Nyarugenge district in Kigali, Rwanda which was less than 24% and a study among women attending health facilities in Kayonza district, Rwanda 28% [33, 34]. This difference is mainly due to differences in the composition and educational level of the participants.

The study has found out that the knowledge level of female healthcare professionals about risk factors of breast cancer was low, with median score of 46.2%. This result is comparable with other similar studies [17, 18]. The main risk factors unknown by the female healthcare professionals were nulliparity, old age at first pregnancy, early menache, and late menopause. Even though it is difficult to control these risk factors, women should be aware of these homone related risk factors in order to teach the general public.

Attitude is a key factor in influencing the health behaviours. Almost half of the participants mentioned that they are unable to detect a breast mass by themselves using BSE. This showed low self-efficacy of the study participants in practing BSE. This might be due to low level of knowledge and inadequate training observed in the study. Self-efficacy is one of the predicator variables to practice BSE [35].

The study found out that there was no statistically significant association between the overall total knowledge score and BSE practice among female healthcare professionals. This finding was in agreement with a prior study conducted among female healthcare professionals in Nigeria [31]. However, knowledge of BSE and BC was significantly associated with BSE practice in studies conducted among healthcare professionals in Ethiopia [19, 27, 36] and Turkey [37]. This finding supports the idea that knowledge doesn’t necessarily change the persons’ health behaviors.

Female doctors had statistically significant higher knowledge level than nurses and other healthcare professionals with median score of 74.3% compared to 62.9% for nurses and 58.6% for other professions. This finding is supported by prior studies conducted in Morocco among healthcare professionals [32]. The knowledge level of female healthcare professionals in this study was much higher than a study done in Saudi Arabia [17]. This might be due to lack of updated courses and focus on BC.

Female healthcare professionals who received training on BSE on either the job or school had higher knowledge score than those without training. However, it is only one quarter of the study participants claimed to attend the training on BSE. This might be due to less focus given in the curriculum of nursing. To go into the cause of low training attendance, further studies with qualitative approach is required.

Holders of Bachelor degree (A0) had statistically significant higher total knowledge score than advance diploma (A1). This is in consistent with a prior study done in Oromia region of Ethiopia [27].

Participants’ attitude towards BSE and BC was significantly associated with BSE practice. This finding was supported by prior studies done in Turkey [38]. However, this is in contrary to the results obtained in studies carried out in Nigeria [32] and Morocco [32] where attitude was not associated with practice.

There was statistically significant differences in the total attitude scores among the different professions. Doctors had a higher attitude score than nurses and other professions with median scores of 86.8, 72.6, and 73.7% respectively. However, the attitude score didn’t differ significantly among the different level of education.

The multivariate analysis has shown that attitude towards BSE and BC as the only significant predictor variable to perform BSE. This finding was supported by prior studies done in Ethiopia [19].

In addition, knowledge and attitude had a positive linear relationship with r = 0.186, *p* = 0.005.

There were certain limitations in the study. The first was the practice of BSE was assessed by self-reporting by the respondents. This might not provide the actual facts as some respondents might not adequately remember the timing and frequency of practice. The second limitation was that respondents were female healthcare professionals working in the district hospitals, this result may not reflect those working in the health centers and private institutions. Despite this limitation, the study identified important gaps in knowledge and practice of BSE among healthcare professionals in Kigali, Rwanda.

## Conclusions

The median knowledge and attitude scores of the participants were 62.9 and 73.7% respectively. Moreover, only less than half (42.53%) of the female healthcare professionals practiced BSE. The main reason for not practicing BSE was fear of being diagnosed with breast cancer. Attitude was the only predictor for BSE practice that was statistically significant.

The study recommends the Ministry of Health and Rwanda Biomedical Center to organize trainings for healthcare professionals who are considered as role models for the public to fill the knowledge gap and promote early detection of breast cancer among the health professionals and in the society at large. The study suggests for further research to be carried out that involves both quantitative and qualitative approaches in order to understand the causes for low knowledge and prevalence of BSE practice among healthcare professionals.

## Data Availability

The dataset used and analyzed for the current study is not publicly available because we are planning to produce other papers. However it is available from the corresponding author on reasonable request.

## Abbreviations

ACS: American Cancer Society
BC: Breast Cancer
BSE: Breast Self-Examination
CBE: Clinical Breast Examination
CI: Confidence Interval
IARC: International Agency for Research on Cancer
RMH: Rwanda Military Hospital
SPSS: Statistical Package for Social Sciences
SSA: Sub-Saharan Africa
WHO: World Health Organization.
